# Medical students' preferences for problem-based learning in relation to culture and personality: a multicultural study

**DOI:** 10.5116/ijme.558e.6451

**Published:** 2015-07-19

**Authors:** Are Holen, Kedar Manandhar, Devendra S. Pant, Biraj M. Karmacharya, Linda M. Olson, Rajendra Koju, Dil I. Mansur

**Affiliations:** 1 Norwegian University of Science and Technology (NTNU), and St. Olav University Hospital, Norway; 2 Kathmandu University School of Medical Sciences (KUSMS), and Dhulikhel Hospital, Kath-mandu University Hospital, Nepal; 3 University of North Dakota School of Medicine and Health Sciences (UNDSMHS) at Grand Forks, North Dakota, USA

**Keywords:** Small group teaching, interactive skills, student satisfac-tion, learning styles, PBL

## Abstract

**Objectives:**

The aim of this study was to explore positive and negative preferences towards problem-based learning in relation to personality traits and socio-cultural context.

**Methods:**

The study was an anonymous and voluntary cross-sectional survey of medical students (N=449) in hybrid problem-based curricula in Nepal, Norway and North Dakota. Data was collected on gender, age, year of study, cohabitation and medical school. The PBL Preference Inventory identified students' positive and negative preferences in relation to problem-based learning; the personality traits were detected by the NEO Five-Factor Inventory. The determinants of the two kinds of preferences were analyzed by hierarchical multiple linear regressions.

**Results:**

Positive preferences were mostly determined by personality; associations were found with the traits Extraversion, Openness to experience, Conscientiousness and Neuroticism; the first three are related to sociability, curiosity and orderliness, the last, to mental health. The learning environments of such curricula may be supportive for some and unnerving for others who score high on Neuroticism. Negative preferences were rather determined by culture, but also, they correlated with Neuroticism and Conscientiousness. Negative preferences were lower among females and students living in symmetrical relationships. Some high on Conscientiousness disliked group work, and the negative correlation with Agreeableness indicated that less sociable students were not predisposed to this kind of learning activity.

**Conclusions:**

Preferences related to problem-based learning were significantly and independently determined both by personality traits and culture. More insights into the nature of students' preferences may guide aspects of curriculum modifications and the daily facilitation of groups.

## Introduction

Towards the end of the last millennium, there were several reasons to reform medical curricula. One was the gap between the goals stated on paper and the achievement in practice of the knowledge, skills and attitudes of medical students. Additionally, the speed of incoming new scientific discoveries, advanced diagnostics, therapeutic technologies, and the availability of medical knowledge via Internet exerted new demands on the health programs.^1^^,^^2^ Modern society emphasizes ethical, egalitarian and democratic social exchange, also between health professions and patients. Related skills and attitudes are more likely to develop in interactive group settings. Problem-based learning (PBL) has increasingly been adopted by medical schools worldwide in response to The Edinburgh Declaration of 1988 and the follow-up conference in 1993.^3^^,^^4^ In PBL, the problem drives the learning in small groups facilitated by tutors.^5^ Research indicates that PBL may be superior to traditional teaching in fostering understanding and the retention of knowledge,^6^ but also in developing social and cognitive abilities.^7^

The challenge in PBL is to make the small groups function effectively. Understanding the contributing elements to good and goal-oriented group dynamics is therefore relevant. To enhance the breadth of the students' learning and to improve the quality of the physicians of the future, topics such as learning styles,^8^ ethics, professionalism,^9^^,^^10^ and the doctors' roles and identity^11^ have been introduced by medical educators. In another field, research on personality traits has made considerable advances in recent decades. To a limited degree these insights have been explored within medical education. Doherty & Nugent (2011) published a literature review of personality and medical training, but did not correlate their findings with the students' attitudes towards PBL.^12^ Bigsby et al. (2013) studied personality with the main focus on learning styles.^13^ Lievens et al. (2002) used the five-factor model to predict medical students' academic performance, but did not relate it to PBL.^2^^,^^14^ Some medical schools interview their applicants;^15^ the underlying assumption is that relevant traits can be identified and used for the selection of good medical students as well as good physicians, later on.

Today, the five-factor model "The Big Five" and the derived inventories are regarded as the most advanced approach to measure personality traits. These inventories have good psychometric properties and have been widely used, and they have proven to be valid and reliable across cultures.^16^ While at the crossroads between personality traits and PBL, medical schools' may gain insights for the development of knowledge, professional attitudes and social, collaborative skills in their curricula.

In the interactive groups of PBL, students are expected to take more responsibility for their learning. Accordingly, their motivation, attitudes, likes and dislikes, in short, their preferences towards the small group activity will sway the upshot of their work, more so than in lecture or seminar settings. Thus, the students' preferences are likely to be important determinants of the outcome of the PBL work. PBL groups have the potential to provide more than cognitive learning; they may promote team work skills and personal development.^17^ The social potentials of PBL groups in developing social, collaborative skills, ethical standards and professionalism are, however, often overlooked. Holen has suggested that shared self-reflections and constructive peer feedback should be made a formal part of PBL.^18^ In leadership training, or in inter-disciplinary collaboration, health professionals are often involved in receiving and giving feedback. The development of such reflecting interpersonal skills should not solely be left to the individual, but aided towards higher levels of sophistication from early on in medical school. PBL groups offer suitable learning environments for such aims.

To date, measures of medical students' positive and negative preferences or attitudes towards the key features of PBL are found lacking. For this purpose, an inventory originating from Norway was tested and eventually used in an international context in this study. The assumption is that the PBL preferences express student motivation, likes and dislikes or attitudes towards central features of PBL, and that these student preferences are reflected in the quality and outcome of the group work. Furthermore, personality is assumed to be a major contributor to the students' preferences.

In addition, socio-cultural factors are assumed to play a role in shaping the PBL preferences. For this reason, representative samples of medical schools from three different continents were sought. Socio-cultural parameters in this study were such as the gender distribution of the medical schools, the pattern of cohabitation of the medical students during their study years, possibly also the age of the students and their year of study, in addition to the medical school itself. Below we present briefly some characteristics of the three medical schools involved in the study. They all have PBL as a central component in the initial years of hybrid curricula, and the student participation in the PBL groups is obligatory.

### Medical schools

In Nepal (KUSMS), PBL was introduced in 2001. The curriculum consists of four and a half years followed by internship of one year. PBL groups of seven or eight students are held on pre-clinical topics the first two years with one new case every week. The PBL sessions last two hours, and are held three times a week on alternative days. After the PBL session, students are provided two-hours to prepare their learning objectives. At the end of each block, there is a two-hour wrap-up session.

In Norway (NTNU), PBL was introduced in 1993. The curriculum lasts six years followed by internship of one and a half years. PBL groups include eight students. In the first two years, PBL groups of two hours duration are held twice a week, while in the third and fourth year, PBL groups meet once a week for three hours. One new case is introduced at every PBL session.

In North Dakota (UND), PBL was introduced in 1998. The medical curriculum consists of four years; PBL covers the first two years. Seven or eight students meet for two hours on alternative days, three times weekly. One new patient case study is introduced each week; the case is concluded with a physician-patient wrap-up session at the end of the week.

### Objective

The purpose of this research study was to examine whether there are relationships between personality traits, culture and preferences for a certain approach to medical education, namely hybrid problem-based learning curricula. We have postulated that medical students' likes and dislikes, attitudes or preferences for PBL are not only linked to their personality traits, but also to their socio-cultural context. If these assumptions are confirmed, the findings may be useful for planning, or modification of medical education curricula, and also, they may have the potential to inform PBL tutors about students' contributions to the effectiveness of the small groups. To pursue these notions, our study explored positive and negative PBL preferences in countries as socio-culturally apart as Nepal in Asia, Norway in Europe and North Dakota in North America. The aim of this study was to explore possible links between the students' PBL preferences and personality in the context of diverse socio-cultural backgrounds.

## Methods

### Design and Procedure

This is a cross-sectional survey. In North Dakota and Nepal, the distributed survey was written in English, which is the teaching language at both institutions. At the NTNU, the survey was in Norwegian. In all three locations, one or two classes were approached and asked to participate in an international survey on PBL preferences. Anonymous consent was used, and the students' were free to participate. No student's name was ever registered or associated with the completed questionnaires at any site. On our request, the REC Central Norway (Regional Committee for Medical and Health Research Ethics) exempted the study from collecting written informed consent due to the anonymous nature of the data collection.

### Participants

Data was collected in 2011 and 2012 from three medical schools: from Kathmandu University School of Medical Sciences (KUSMS), Nepal, 123 students (65% participation from the local class; 27.4% of the total sample studied); Norwegian University of Science and Technology (NTNU), Norway, 229 students (67% local class participation; 51.0% of total); University of North Dakota School of Medicine and Health Sciences at Grand Forks (UNDSMHS), USA, 97 students (75.5% local class participation; 21.6% of total), in total, 449 (100%) participants.

### Socio-cultural information

Data was collected on the medical students' gender, age, cohabitation, medical school and the year of study. Age was used as a continuous variable; though a note was added in the survey to assure anonymity: "You may drop this [item about age] if you find that it may identify you". Fifty-two (12% of total) students used this response option. The four categories of cohabitation are displayed in Table 1. Most participants had about one year or more of PBL experience when the survey was completed. In Nepal, most students were in their 2nd year, but some were in their internship (6th year; N=32). In Norway, the students were all in their 2nd year. In North Dakota, data was collected from one class at the end of the first year (N=55) and from another at the start of their second year (N=42).

### PBL Preference Inventory (PPI)

This inventory has been developed and administered at NTNU by the first author using reiterative rewordings of the items over several years, always with 2nd year students, until a good fit was achieved by factor analyses with the principal component extraction method and varimax rotation. In PPI, the students are asked to indicate how true each of six items are to them on a scale from 0 to 9; 0 - "Not at all", 2 - "A little", 4 - "Moderately", 6 - "Much", and 8 - "Very much"; the response options 1, 3, 5, 7, 9 could be used to modify their responses up or down.

**Table 1:** Socio-cultural information by survey from three medical schools compared (N=449)

Variable		Nepal N=123F (%)	Norway N=229F (%)	N. Dakota N=97F (%)	p-value
Gender					
	Females	42(34, 9)	127(55, 28)	45(46,10)	Chi sq.=15.92;p<0.001
	Males	81(66,18)	98(43,22)	51(53,11)	
	Missing	0(0, 0)	4(2, 1)	1(1, 0.2)	
Age					
	Mean	21.4	22.1^a^	24.1^b^	F=50.56;p<0.001*
	Non-responders	15(12,3)	21(9, 5)	16(16, 4)	
Cohabitation					
	Living alone	4(3, 1)	40(17, 9)	34(35, 8)	Chi sq. =125.14;p<0.001
	With parents, relatives, friends of parents	31(25, 7)	8(3, 2)	2(2,0.4)	
	With fellow students	88(72, 20)	126(55, 28)	30(31,7)	
	With partner, spouse and/or children	0(0, 0)	45(20, 10)	28(29, 6)	
	Missing	0(0, 0)	10(4, 1)	3(3, 1)	
Year of study					
	1^st^ year, (N Dakota)	0(0)	0(0)	55(57,12)	Chi sq. = 312.50;p<0.001
	2^nd^ year	91(74, 20)	229(100,51)	42(43, 9)	
	6^th^ year (Nepal)	32(26,7)	0(0, 0)	0(0, 0)	

a sign. diff. Norway > Nepal; p=0.03. b sign. diff. N Dakota > Norway & Nepal; p=0.00. *no significance

In this study, the PPI was used in English for the first time. Accordingly, the double back translation method was applied in the development of the English version. To ascertain that the inventory in the study had a factor structure that was independent of the location of the respondents, factor analyses with the principal component extraction and varimax rotation was carried out within the sample of each medical school, and also for the total population. The two-factor solution with the same item distribution appeared in the samples from Nepal and North Dakota as in the original Norwegian version, and also in the total sample. The inventory absorbed 63.6% of the total variance (N=449). The two-factor solution absorbed 68.0% in Nepal (N=123), 59.3% in Norway (N=229), and 60.0% in North Dakota (N=97).

According to the item content, the first factor of the total population reflected the negative and the second factor positive PBL preferences of the students. The two factors were converted into two separate scores, each consisting of three items. One item had high loadings on both factors; due to the semantic item content and the negative correlation with PBL Negative, it was assigned to the PBL Positive. The mean sum of the non-weighted items no. 3, 5, 6 gave the PBL Negative score (M 3.04 (SD 1.95); Cronbach alpha = 0.65; item-to-sum correlations ranged from 0.72-0.80). Items no. 1, 2, 4 gave the PBL Positive score (M 6.35 (SD 1.27); Cronbach alpha = 0.64; item-to-sum correlations ranged from 0.73-0.80). No item deletion would give a higher Cronbach alpha value for any of the two scores. The two factors and their related scores do not represent a continuum. The correlation between PBL Positive and PBL Negative was negative (r= -.36; p< .001). For more details about PPI, the item distribution and the related statistics, see Table 2.

**Table 2:** Factor analysis of items related to PBL preferences from survey*(N=448)

To what extent...	F1	F2
1. Do you like PBL as a learning method?	-0.67	0.48
2. Do you participate actively in PBL groups?		0.82
3. Would you rather replace PBL groups in the curriculum with lectures?	0.84	
4. Are you usually well-prepared for the PBL groups?		0.84
5. In your opinion, do you think that much time is wasted in PBL groups?	0.84	
6.Do you dislike evaluations and feedback of your personal contributions to PBL groups?	0.56	

### NEO-FFI (60-item version)

Personality traits were captured by the NEO Five-Factor Inventory (NEO-FFI); the short version of 60 items was chosen.^19^ The respondents indicate how true each item is for them by scoring 0-4; 0 - "Not true" 2 "Somewhat true" and 4 "Very true". The inventory has been thoroughly validated in English^19^ and in Norwegian.^20^ The five personality traits are Neuroticism (M 1.56; SD 0.74), Extraversion (M 2.69; SD 0.57), Openness to experience (M 2.29; SD 0.46), Agreeableness (M 2.75; SD 0.54), and Conscientiousness (M 2.87; SD 0.53). The mean values (M) and the standard deviations (SD) given here are from this study.

### Statistics

The data were analysed in two steps in relation to the PBL Positive and PBL Negative as the dependent variables, first by bivariate correlation analyses, thereafter by hierarchical multivariate, linear regression analyses. In the bivariate correlation analyses, Pearson's Chi squared was used between two categorical variables; One Way ANOVA with Scheffe's procedure was applied whenever continuous variables were explored in relation to the categorical variables with three or more response options. Pearson's correlation was used for two continuous variables.

In the second step, hierarchical regression analyses were carried out to explore the associations of PBL Positive and PBL Negative as dependent variables. For both, an identical set of independent variables consisting of three blocks were entered: the first covered the socio-cultural variables; the second encompassed the five personality traits, and since there was some overlap between the two preferences, the last block contained the opposite PBL preference. Categorical variables were re-coded into dummy variables.

SPSS version 19 was used in all the statistical computations.^21^ Statistical significance was decided whenever p<0.05, and no p-value was reported as less than p<0.001. For missing values, this principle was followed: when one item was not endorsed for any of the two PPI scales, or when 1-2 items from any of the NEO-FFI scales, the missing values were replaced by mean values. However, if more items were missing in the composite variables, those participants were excluded from the computations. Missing information about age was replaced with mean values.

## Results

Gender, age and cohabitation as well as aspects of the medical schools were unevenly distributed between the three medical schools, see Table 1.

In the bivariate correlations with PBL Positive and PBL Negative, no significant gender difference was found. A significant correlation (r=0 .11; p< 0.05) was found for age with PBL Positive, but not for PBL Negative. PBL Positive was scored higher by those who lived with partners, spouses and/or children. Students from North Dakota scored significantly higher on PBL Positive than those from Nepal and Norway. Students from NTNU scored significantly higher on PBL Negative than those from Nepal and North Dakota. With regard to personality, all traits correlated significantly with the PBL Positive; Neuroticism had a negative correlation. For PBL Negative, only Openness to experience correlated significantly and negatively.

In both regression analyses, three identical blocks of independent variables were used in relation to the two preferences. The independent variables of each block are displayed in detail in the tables 3 and 4. When using PBL Positive as the dependent variable, gender, age and cohabitation played no significant role. The medical school in Nepal showed a significant negative correlation. Entering the five personality traits elevated the adjusted R^2^ =0.07 (F=3.98; p<0.001) to R^2^ =0.20 (F=7.37; p<0.001). Extraversion, openness to experience, conscientiousness and neuroticism emerged with significant positive correlations with PBL Positive. PBL Negative showed a significant negative correlation with PBL Positive and elevated further the adjusted value to R^2^=0.31 (F=12.28, p<0.001). See Table 3.

**Table 3:** PBL Positive preference from the PBL Preference Inventory as dependent variable in hierarchical linear regression analysis with three blocks of independent variables from survey (N=436)

Three blocks of independent variables	Adjusted for socio-cultural information			Adjusted for personality traits			Adjusted for PBL negative preference		
	Β (standardized coefficient)	t	p-value	Β (standardized coefficient)	t	p-value	Β (standardized coefficient)	t	p-value
Block 1: Socio-cultural info									
(Constant)		6.98	0.001		2.65	0.008		4.81	0.001
Gender (1: Male, 2: Female)	0.01	0.26	0.797	-0.04	-0.74	0.458	-0.08	-1.58	0.114
Age	-0.08	-1.15	0.250	-0.06	-1.01	0.312	-0.07	-1.27	0.204
Cohabitation									
Living alone	0.05	042	0.679	0.07	0.60	0.550	0.01	0.11	0.914
With parents etc.	-0.07	-065	0.518	-0.64	-0.67	0.502	-010	-1.17	0.245
Fellow students	0.09	0.56	0.579	0.09	0.62	0.538	-0.03	-0.25	0.805
Partner / children	0.12	1.01	0.315	0.10	0.85	0.397	-0.001	-0.009	0.993
Medical school info									
1^st^ Year	-0.05	-0.67	0.504	-0.04	-0.55	0.583	-0.08	-1.20	0.229
2^nd^ Year									
6^th^ Year	0.10	1.57	0.117	0.09	1.66	0.098	0.16	2.92	0.004
Nepal	-0.12	-1.88	0.060	-0.06	-0.98	0.328	-0.29	-4.36	0.001
Norway									
N Dakota	0.27	3.39	0.001	0.21	2.82	0.005	0.12	1.72	0.086
R^2^ adjusted	0.07								
F of equation	F= 3.98; p<0.001								
Block 2: Personality traits									
Neuroticism				0.08	1.52	0.129	0.13	2.47	0.014
Extraversion				0.24	4.35	0.001	0.18	3.56	0.001
Openness to experience				0.16	3.39	0.001	0.11	2.59	0.010
Agreeableness				-0.05	-0.84	0.403	-0.09	-1.70	0.090
Conscientiousness				0.25	5.04	0.001	0.28	6.00	0.001
R^2^ adjusted				0.20					
F of equation				F= 7.37; p< 0.001					
Block 3: Opposite PBL preference									
PBL Negative							-0.40	-8.17	0.001
R^2^ adjusted							0.31		
F of equation							F=12.28; p<0.001		

With PBL Negative as the dependent variable, gender showed a significant negative correlation. Age did not reach significance. Students who lived with fellow students or a partner, spouse and/or children, had significant negative correlations. Moreover, students from Nepal and North Dakota were significantly and negatively correlated with PBL Negative. The first block consisting of the socio-cultural variables appeared with an adjusted value to R^2^ =0.20 (F=10.73; p<0.001). Personality traits significantly associated with PBL Negative included Neuroticism and Conscientiousness, while Agreeableness was negatively correlated. The personality traits elevated the adjusted value to R^2^ =0.26 (F=10.03; p<0.001).  PBL Positive was negatively associated, and elevated the adjusted value to R^2^=0.37 (F=15.20; p< 0.001). See Table 4.

**Table 4:** PBL Negative preference from the PBL Preference Inventory as dependent in hierarchical linear regression analysis with three blocks of independent variables from survey (N=436)

Three blocks of independent variables	Adjusted for socio-cultural information			Adjusted for personality traits			Adjusted for PBL negative preference		
	Β (standardized coefficient)	*t*	p-value	Β (standardized coefficient)	*t*	p-value	Β (standardized coefficient)	*t*	p-value
Block 1: Socio-cultural info									
(Constant)		4.21	0.001		4.99	0.001		6.46	0.001
Gender (1: Male, 2: Female)	-0.12	-2.63	0.009	-0.09	-1.87	0.062	-0.11	-2.34	0.020
Age	-0.04	-0.59	0.559	-0.03	-0.42	0.677	-0.05	-0.88	0.382
Cohabitation									
Living alone	-0.07	-0.64	0.523	-0.14	-1.29	0.200	-0.12	-1.14	0.255
With parents etc.	-0.06	-0.64	0.522	-010	-1.05	0.296	-0.12	-1.42	0.158
Fellow students	-0.26	-1.77	0.078	-0.31	-2.18	0.030	-0.27	-2.11	0.036
Partner / children	-0.21	-1.85	0.065	-0.24	-2.21	0.028	-0.20	-2.04	0.042
Medical school info									
1^st^ Year	-0.09	-1.35	0.177	-0.10	-1.46	0.145	-0.11	-1.81	0.071
2^nd^ Year									
6^th^ Year	0.15	2.68	0.008	0.15	2.76	0.006	0.19	3.67	0.001
Nepal	-0.46	-7.98	0.001	-0.56	-8.91	0.001	-058	-10.05	0.001
Norway									
N Dakota	-0.25	-3.36	0.001	-0.23	-3.14	0.002	-0.15	-2.19	0.029
R^2^ adjusted	0.20								
F of equation	F=10.73; p<0.001								
Block 2: Personality traits									
Neuroticism				0.11	1.99	0.047	0.14	2.79	0.006
Extraversion				-0.14	-2.68	0.008	-0.05	-1.05	0.293
Openness to experience				-0.11	-2.53	0.012	-0.06	-1.29	0.196
Agreeableness				-0.11	-1.91	0.057	-0.12	-2.42	0.016
Conscientiousness				0.07	1.33	0.184	0.16	3.44	0.001
R^2^ adjusted				0.26					
F of equation				F=10.03; p<0.001					
Block 3: Opposite PBL preference									
PBL Negative							-0.37	-8.17	0.001
R^2^ adjusted							0.37		
F of equation							F=15.20; p<0.001		

## Discussion

The study shows that personality traits play a major part in determining medical students' positive PBL preferences. The same is true, but to a much lesser extent for the PBL negative preferences where socio-cultural dimensions play a more substantial role. Independent of their whereabouts in the world, the preferences, likes or dislikes of medical students towards PBL are to a significant degree related both to personality and to the cultural context of the medical student. The findings met our main expectations, and they may have relevance for several types of interactive small group learning settings. The findings about the five personality traits and the two PBL preferences seem to point in the same direction. PBL is appreciated by outgoing, curious, sociable and conscientious students, and to some extent, also by some, but not all students high on neuroticism. The most relevant traits for medical education found in other studies have been conscientiousness, extraversion and openness to experience.^22^ Of the socio-cultural dimensions, females, but also students who live with other fellow students or with their partners or spouses, i.e. companionable participants who have chosen to live in fairly symmetrical social relationships, demonstrate less negative PBL preferences. This is in contrast to the students who live with their parents, relatives, or friends of parents, i.e., those who live in asymmetric relationships; the latter tend to be less in favour of PBL. The negative correlation of agreeableness to the negative PBL preference suggests that students low on gregariousness, low on friendly adaptive behaviour and social commitment, but also those not adverse to interpersonal conflicts seemingly have less liking for PBL. Agreeableness involves being cooperative, nurturing, affectionate, and sensitive, but also having a dislike for conflict and interpersonal confrontation.

The gender distribution of the three medical schools plausibly exhibits societal attitudes towards females entering higher education. The students' cohabitation patterns may also reflect societal differences and norms. The higher mean age of students from the US is likely related to the college graduation required before entering medical school. In Norway and Nepal such demands do not exist, and students will normally start at a lower age at medical school. Age played no role in relation to any of the PBL preferences when other factors were corrected for, while the liking of PBL may perhaps increase with the years in PBL learning environments.

Negative PBL preference is explained far more by the socio-cultural background of the student. In relation to this preference, the inclusion of the block with personality traits made a minor, yet significant addition to the explained variance. Students from Nepal were less enthusiastic about PBL than students from North Dakota; the non-authoritarian and self-administered learning format of PBL may be culturally unfamiliar to some Nepalese. In PBL curricula, the students' knowledge, self-discipline, and interpersonal skills are continuously exposed to fellow students and tutors. This transparency requires social trust and provides less opportunity to keep a social distance and for hiding one's ways. For most, to be seen and to be socially involved may gratify proximity needs and induce a sense of mutual social commitment. For a few, however, the transparency, the high level of social and academic exposure and the obligations towards the PBL group may be met with reluctance, perhaps even activate paranoid fears, or it may seem intimidating for the socially inhibited. In both cases, PBL may be felt as intrusive and prying. In traditional curricula, students have more "freedom" to hide. They can choose if, how and when they want to learn; they can easily remain unexposed on the backbenches of the lecture halls.

Neuroticism has a positive association with both preferences. The trait reflects a general propensity to respond to events, circumstances and challenges with dysphoric affect and apprehension, sometimes even with unjustified suspicion. The social inclusion and interactive nature of PBL groups may offer a supportive environment for some socially insecure students high on Neuroticism; they may relax and feel integrated into the small groups. However, other socially anxious or reticent students may feel uneasy and uncomfortable in the closely-knit PBL groups; they may feel untroubled with more social distance and prefer impersonal learning arenas rather than the small group settings. The two opposite responses to the social proximity of the PBL groups may explain the dual relationship of Neuroticism in relation to the PBL preferences.

Conscientiousness seems to be a significant predictor of academic success,^2^^,^^12^^,^^14^ but also of adequate group behaviour.^23^ Worth noting are the findings of Lievens et al. concluding that conscientiousness is important in predicting long-term success in medical training. In the same way as for neuroticism in our study, both PBL preferences were positively associated with conscientiousness.2 A separate study has related higher scores on conscientiousness to compulsiveness,^24^ i.e., a marked, sometimes even an obsessive need for order and structure. To some extent, this trait may explain why some students high on conscientiousness may dislike PBL; the interactive free flow of the small groups may be regarded as an unruly chaos.^25^

This study is the first to link students' attitudes or preferences for PBL to personality traits. The findings concur in part with a study that compared students on a traditional lecture-based track with students on a PBL track; those with favourable attitudes towards psychiatry demonstrated more "Openness to experience" and "Agreeableness".^26^ The interpersonal involvements of PBL probably improve social skills as emphasized by Koh’s meta-study.^7^

As of yet, personality traits have not been specifically linked to the ideal doctor, but a set of roles has been discussed in this regard.^27^^,^^28^ Roles are generally seen as sets of behaviours that can be learnt independently of personality. Even so, it can be assumed that some roles would more readily be displayed by persons with certain personality profiles. To work as a physician usually involves considerable social interaction. Some tend to feel more at ease in asymmetrical, i.e., in the traditional doctor-patient relationships with the physician in the one-up position. It requires far more social skills of the doctor to engage in fairly symmetrical professional relationships with patients. PBL seems to be liked by students who prefer symmetrical relationships. Also, PBL is presumptively more fitted for reinforcing and developing symmetrical interpersonal skills as a preparation for the students' work in the PBL groups, and also, towards their subsequent professional interaction. However, in certain areas of medicine, social skills matter less.

From an ethical point of view, the contention that PBL favours one set of personality traits, may be viewed as discrimination against those who otherwise qualify for admission. However, literature lacks studies indicating that lecture-based teaching enhances personality traits that would be more favourable for physicians. Several studies indicate rather the opposite.^7^

It has been argued that students in their early stages of medical school tend to be more inclined towards being competitive and impersonal.^29^^,^^30^ Research findings by Chibnall, Blaskiewics & Detrick showed that agreeableness was not a strong suit of medical students in the initial part of the study.^31^ Curricula that early on modify students' behaviour towards symmetrical interaction as in PBL are probably bringing the students more in line with the expectations of the modern sophisticated patients of today and the future. The negative correlation between the two PBL preferences suggests that some students may like both aspects of their hybrid curricula, the PBL groups as well as the lectures. A few students may even be dissatisfied with whatever curriculum they would be involved in.

### Limitations and strengths

In general, survey data tend to have lower reliability and validity than interview data.^32^ The study shows mostly how PBL is seen at an early stage of the medical curriculum, and not how the attitudes towards PBL may change in the later stages or even after the completion of medical school. Likewise, the cross-sectional survey cannot be regarded as a too reliable source for how the PBL preferences may develop over time. There are some vague indications that the first year students from North Dakota favour PBL less so, and 6th year students from Nepal regard PBL more highly than the second year students, but the data does not necessarily concur with firm conclusions about this. The study's level of student participation within each site is about two-thirds of the total classes, which is acceptable. A higher participation would have reduced the weight of any possible self-selected bias in the study.

The PPI inventory is new and brief; it has been standardized over years on Norwegian medical students in their second study year. This is the first time IPP is used in an international context. The psychometric properties within each country are found to be acceptable, and the preference concepts, i.e., the item structure of PBL Positive and PBL Negative of the two factors, are the same across the three locations. Even so, the generalizability of the findings from this new inventory should be handled with some caution until the item structure and the related preference scores have been tested by further studies in diverse contexts. In this study, the concepts implied by the item distribution of the two preferences did not seem to be confined to any particular culture. The same factor structure or item distribution as found in Norway appeared also in the two other medical schools; essential cross-cultural dimensions of the original version have suggestively been safeguarded by the translation. The Cronbach alpha algorithm makes it difficult to obtain high values for scales with less than 10 items. In this study, the number of items is three. Even so, the internal consistency is acceptable. However, it should be kept in mind that with a low number of items, the scale is more sensitive to misunderstandings. The item-to-sum correlations provide a more adequate picture when the number of items is small; the ranges of the correlations were either good or acceptable. PBL is a heterogeneous concept, as pointed out by some researchers.^33^ Differences exist between the three PBL curricula in this study. Nevertheless, they shared several pivotal features: small group learning, problem-oriented case work and lectures.

The strengths of the study lie in the fact that three rather different medical schools and cultures are represented, which helps in the differentiation between personality issues and culture. The number of participants from each site is acceptable; the sample size from each location can be assumed to be fairly representative of their respective classes. The personality measure is solid and well-established with very good psychometric properties and multicultural strengths. The PPI is a new instrument that specifically addresses issues related to PBL, and as such, it may offer new angles for the exploration of small group learning environments. Conceptually, the findings in relation to the two PBL preferences point in the same direction.

## Conclusion

The reforms of recent decades involving the introduction of PBL into medical curricula are generally associated with expectations of improved cognitive and social learning. So far, the body of research within medical education that has utilized the advances of personality trait research has been rather small. The current study demonstrates reasonable connections between students' PBL preferences and their personality traits. Also, the study emphasizes that negative PBL preferences pose more challenges on the socio-cultural level. In modern society, student participation is commonplace in the shaping of academic curricula. To know the students' positive and negative preferences towards any curriculum may turn out to be helpful in developing educational programs. The findings of the present study may also have implications for the day-to-day facilitation in PBL groups.

This study shows that the personality traits extraversion, openness to experience and conscientiousness, but also neuroticism are closely linked to positive preferences for PBL. Some less sociable persons may have compulsive traits and some with mental problems may disfavour PBL. The study does not link personality traits to academic success, but to PBL preferences or student satisfaction. Further investigations of the Big Five traits in relation to PBL and small group learning activities seem relevant for the social and cognitive effectiveness of small groups in academic settings.

### Conflict of Interest

The authors declare that they have no conflict of interest
